# Osteoporosis and gut microbiota: current understandings

**DOI:** 10.3389/fimmu.2026.1761421

**Published:** 2026-02-24

**Authors:** Yikai Liu, Shuai Lu, Ruifeng Bai, Pengli Han, Yejun Zha, Xieyuan Jiang

**Affiliations:** 1 Department of Orthopaedics and Traumatology, Beijing Jishuitan Hospital Affiliated to Capital Medical University, Beijing, China; 2 Beijing Research Institute of Traumatology and Orthopaedics, Beijing, China; 3 Department of Laboratory, Beijing Jishuitan Hospital Affiliated to Capital Medical University, Beijing, China; 4 Pharmaceutical Department, Zhengzhou Central Hospital Affiliated to Zhengzhou University, Zhengzhou, China; 5 Department of Orthopedic Trauma, Zhengzhou Central Hospital Affiliated to Zhengzhou University, Zhengzhou, China

**Keywords:** bone metabolism, gut microbiota, leaky gut, osteoporosis, probiotics

## Abstract

Osteoporosis (OP), a prevalent disease among middle-aged and elderly individuals, is characterized by a decrease in bone mineral density (BMD), which predisposes individuals to fragile fractures. The core pathological process involves excessive bone resorption over bone formation. The etiology of OP is intricate, and increasing recent studies have focused on the role of gut microbiota (GM) in its pathogenesis. Harmful bacteria exacerbate OP, whereas probiotics are beneficial for bone metabolism and delay the progression of OP. GM influences the progression of OP through various mechanisms, including the production of metabolites, modulation of the intestinal barrier, and regulation of intestinal immunity and osteoimmunology. In this review, we summarize the role of GM in the pathogenesis of OP based on the latest understandings, and highlight the promising potential of strategies targeting GM for the intervention and management of OP.

## Introduction

Osteoporosis (OP) is a prevalent systemic skeletal disease among the elderly people, which makes them vulnerable to bone fractures, and poses a great threat to their health. With the progress of the population aging, OP has gradually become a severe health threat and a social problem. Previous studies have indicated that osteoporotic fractures occur every three seconds worldwide and approximately half of all women and one-fifth of men experience an osteoporotic fracture after reaching the age of 50 (1, 2). This highlights the significant impact that OP has on the global population, particularly as people age. Therefore, the prevention and treatment of OP has gradually become a key concern for the health of the elderly people, and reducing the incidence of OP is vital for easing medical burden and improving the quality of life of the population.

The etiology and pathogenesis of OP are intricate and are marked by a delicate imbalance between bone resorption and bone formation. The maintenance of skeletal homeostasis relies on the precise balance between osteoclast mediated bone resorption and osteoblast mediated bone matrix formation, When the rate of bone resorption is faster than the rate of bone formation, the skeletal homeostasis is disrupted, leading to bone loss and OP (3). There are various factors affecting the balance of bone resorption and bone formation, such as aging, the withdraw of estrogen, drugs like corticosteroids, hyperparathyroidism, and immune imbalance in bone microenvironment (4). Several biological processes were involved in the pathogenesis of OP, including cellular senescence, Wnt and OPG/RANK/RANKL (Receptor Activator of Nuclear Factor-B Ligand) are key signaling pathways in bone homeostasis (3, 5). These detrimental factors induce chronic inflammation, disrupt the equilibrium between osteogenesis and lipogenic differentiation of BMSCs (bone marrow stromal cells) (6), and compromise the formation of H-type blood vessels (7). Ultimately, they lead to reduced bone formation and increased bone resorption, culminating in bone loss.

The human gastrointestinal tract is home to over 100 trillion bacterial cells and 3 million genes, forming an extremely large microbial community (8). This microbial community plays a crucial role in maintaining environmental stability within the human body. Alterations in the composition of the gut microbiota (GM) can lead to the development of numerous chronic health issues in humans, such as obesity (9), metabolic disorders (10–12), diabetes mellitus (13), neurological diseases (14), autoimmune diseases (15), tumor (16–18), cardiovascular diseases (19) and musculoskeletal diseases such as osteoarthritis (20), sarcopenia (21–23) and OP (24–27). The past two decades have seen a dramatic surge in interest regarding the role of GM in health and disease (28). There are numerous research studies focusing on GM to explore their effects in the pathogenesis of OP, and GM has started to be considered as a potential therapy for OP.

## Gut-bone axis, the evidence of interaction between GM and OP

Mounting evidence has shown the relationship between GM and bone metabolism, highlighting its potential influence of GM in OP pathogenesis. This is exemplified in exercise interventions, as physical activity could protect OVX (ovariectomy)-mice from bone loss, yet this benefit is abolished when the GM is depleted (29). Consistently, fecal microbiota transplantation (FMT) from exercised donor mice alleviates bone loss in recipient OP mice, which directly implicates the role of GM in the maintain of bone mass (29). Beyond intervention studies, compositional analyses further link specific microbial signatures to OP. A recent comprehensive multi-omics study in a Chinese cohort of peri- and post-menopausal women revealed a negative association between *Bacteroides vulgatus* abundance and bone mineral density (BMD) (30). Similarly, a Mendelian randomization analysis indentified 10 GM taxa associated with OP risk, with five increasing and five decreasing risk (31).

The causal role of GM is further supported by animal models. OVX, a standard model animal for OP, fails to induce bone loss in germ-free mice, which indicates that GM is essential for estrogen deficiency-induced OP (32, 33). Moreover, the high-fat diet-induced OP group showed increased abundance of Firmicutes and decreased abundance of *Patescibacteria*, *Epsilonbacteraeota*, *Actinobacteria*, and *Bacteroidota* (34). Conversely, OP treatments themselves can alter the GM, which highlights the complex interplay between OP treatment and the GM (35). Anti-osteoporotic medications, including vitamin D (36), estrogen (37), and aminobisphosphonates (38), have been shown to influence GM composition (e.g., affecting *Bacteroides* spp., *Klebsiella* spp., and *Blautia* spp.).

Investigations into postmenopausal osteoporosis (PMO) patients reveal the associations between the GM profile and bone health. One study observed reduced abundance and diversity of GM in the feces of PMO patients (39). Further supporting the role of specific taxa, another study found a marked decrease in *Prevotella* spp. in PMO patients and demonstrated that transplanting *Prevotella* spp. into OVX mice effectively prevented bone loss (40). The prevention of OP by menopausal hormone therapy is hypothesized to be mediated, in part, through beneficial alterations in the GM composition (37). However, another study on 58 women reported that no significant changes in GM composition were found 6 months after oophorectomy or after subsequent hormonal therapy, indicating that interindividual differences contribute more to the differences in GM composition than hormonal status (41).

The aforementioned link between the GM and bone, also termed as gut-bone axis, involves various GM-modulating molecules. For example, numerous molecules have influence on bone metabolism and OP development by regulating GM, which reinforce the evidence of gut-bone axis. For example, neuropeptide Y (NPY) aggravates PMO in OVX rats by modulating GM diversity and composition, thereby affecting circulating microbial metabolites (27). In addition, intestinal *Firmicutes* and *Bacteroidetes* play a crucial role in osteoclast differentiation. It also found that their ratio (F/B) differed significantly between OVX and sham-operated mice, proposing it as a potential indicator of OP (42). Mechanistically, *Firmicutes* and *Bacteroidetes* influence bone metabolism by modulating the *de novo* synthesis of glutathione (GSH) via the key enzyme Gclc and by inhibiting mitochondrial biogenesis and ROS accumulation through the CREB pathway (42). Other drugs and compounds that prevent OP progression will be addressed later.

### Changes of GM and intestine during aging and OP

The composition and bacterial abundance of the GM exhibit distinct differences not only between young and old individuals but also between those with OP and healthy individuals. Typically, the GM of elderly individuals shows reduced species diversity and greater inter-individual variability. This age-related dysbiosis is often driven by factors such as decreased digestive efficiency, altered dietary patterns, prolonged medication use, and immunosenescence. Consequently, it is characterized by a notable reduction in beneficial butyrate-producing bacteria (which weakens intestinal barrier function) and an increase in pathogenic Gram-negative bacteria (which promote systemic inflammation). Together, these shifts contribute to compromised tight junction integrity and increased intestinal permeability, potentially exacerbating systemic inflammation and bone resorption (43, 44). Compared to younger adults, the GM of older individuals tends to have a higher abundance of *Bacteroides* spp.*, Eubacteria, and Clostridiaceae* species, while *Bifidobacteria* are relatively reduced (45). The reduction in *Bifidobacteria*, which are involved in maintaining gut integrity and modulating immune responses, may further impair the host’s ability to regulate osteoclast activity.

Studies comparing PMO patients with non-postmenopausal OP individuals reveal significant changes in gut bacterial and fungal diversity, along with altered fecal metabolite profiles (46). These differences suggest that estrogen deficiency, a hallmark of menopause, may directly or indirectly shape GM composition, possibly through changes in bile acid metabolism, mucosal immunity, or gut permeability. For instance, one study reported enrichment of *Romboutsia* spp.*, unclassified Mollicutes*, and *Weissella* spp. in non-postmenopausal OP patients, whereas the PMO group showed higher abundances of *Fusicatenibacter* spp., and *Megamonas* spp (47). Although the exact functional implications of these genera remain to be fully elucidated, Megamonas is associated with carbohydrate fermentation and short-chain fatty acid (SCFA) production, and its shift could influence host energy metabolism and immune modulation in an estrogen-deficient environment.

Even in studies where overall alpha and beta diversity does not differ significantly between OP and non-OP groups, notable differences at the phylum and genus levels persist. For example, OP groups have shown higher levels of *Proteobacteria* (a phylum often linked to inflammatory states) and lower levels of *Synergistota* (48). The increase in Proteobacteria may indicate a state of microbial instability and inflammation, which could promote bone loss through pro-cytokine release. Furthermore, a study utilizing 16S rRNA gene sequencing and an LC-MS-based metabolomics approach to investigate the link between estrogen-deficiency-induced OP and the GM, as well as fecal metabolic phenotype, revealed decreased bacterial richness and diversity in PMO patients (39). This suggests that GM changes in OP are not merely compositional but functional, influencing the host metabolome in ways that may directly affect bone remodeling. Specifically, metabolites like N-acetylmannosamine might interfere with osteoblast function or promote osteoclastogenesis, though the precise pathways require further investigation.

Importantly, specific bacterial genera enriched in OP, such as *Klebsiella* spp. and *Allisonella* spp., have shown positive correlations with bone turnover markers like N-terminal propeptide (P1NP) and C-terminal telopeptide of type I collagen (CTX-1). This correlation hints at a direct microbial role in modulating host bone metabolism, possibly through bacterial-derived metabolites that enter systemic circulation and influence osteoclast or osteoblast activity. Although no significant correlation was found between the GM diversity and estrogen levels in that study (39), it remains plausible that estrogen loss alters the gut environment, selecting for bacteria that thrive in a low-estrogen setting and produce metabolites detrimental to bone health.

Finally, even among young individuals, variations in GM composition across different BMD levels have been observed. A study involving 62 Chinese Han youth participants, revealed significant variations in microbial richness and composition among individuals with low, medium, and normal BMD (49). The predominant phyla of GM in this cohort were *Bacteroidota*, accounting for 50.6%, and Firmicutes, comprising 41.6% (49). This implies that GM influence on bone is not limited to aging or estrogen deficiency but may be a lifelong modulator of bone homeostasis, interacting with diet, genetics, and immune function to shape fracture risk over time.

## How does GM affect the pathogenesis of OP

### Metabolites

Extracellular vesicles derived from the GM of children could maintain bone mass and enhance bone strength (50), which indicated that GM prevents OP via their secreted metabolites, at least to some extents. In fact, GM produce several metabolites, such as short chain fatty acids (SCFAs), Butyrate, amino acids, hydrogen sulphide (H2S), bile acids (BAs) and secondary bile acids (sBAs) such as deoxycholic acid (DCA), ursodeoxycholic acid (UDCA) and lithocholic acid (LCA) (17, 51). SCFAs are mainly originated from the anaerobic fermentation of fiber by GM, and including the phyla *Firmicutes* (such as *Clostridium* spp. and *Lactobacillus* spp.), *Actinobacteria* (including *Propionibacterium* spp. and *Bifidobacterium* spp.), *Proteobacteria*, and *Verrucomicrobia*, as well as the genus Bacteroides spp (52). The three primary SCFAs found in the intestines are butyrate, propionate, and acetate; butyrate is mainly produced by *Firmicutes*; propionate by *Firmicutes*, *Bacteroides* (including *Acetobacterium* spp., and the species *Clostridium aceticum*), whereas acetate is produced by *Propionibacterium* spp (53). Bacteria such as *Roseburia* spp., *Faecalibacterium prausnitzii*, and *Coprococcus* spp. can catalyze the condensation of acetate into butyrate in the human colon through the action of butyryl-CoA CoA-transferase (54). DCA, UDCA, and LCA are three main sBAs produced by GM. DCA and LCA are mainly produced by *Clostridium* spp., and UDCA is mainly produced by Parabacteroides distasonis (55, 56).

**Figure 1:**
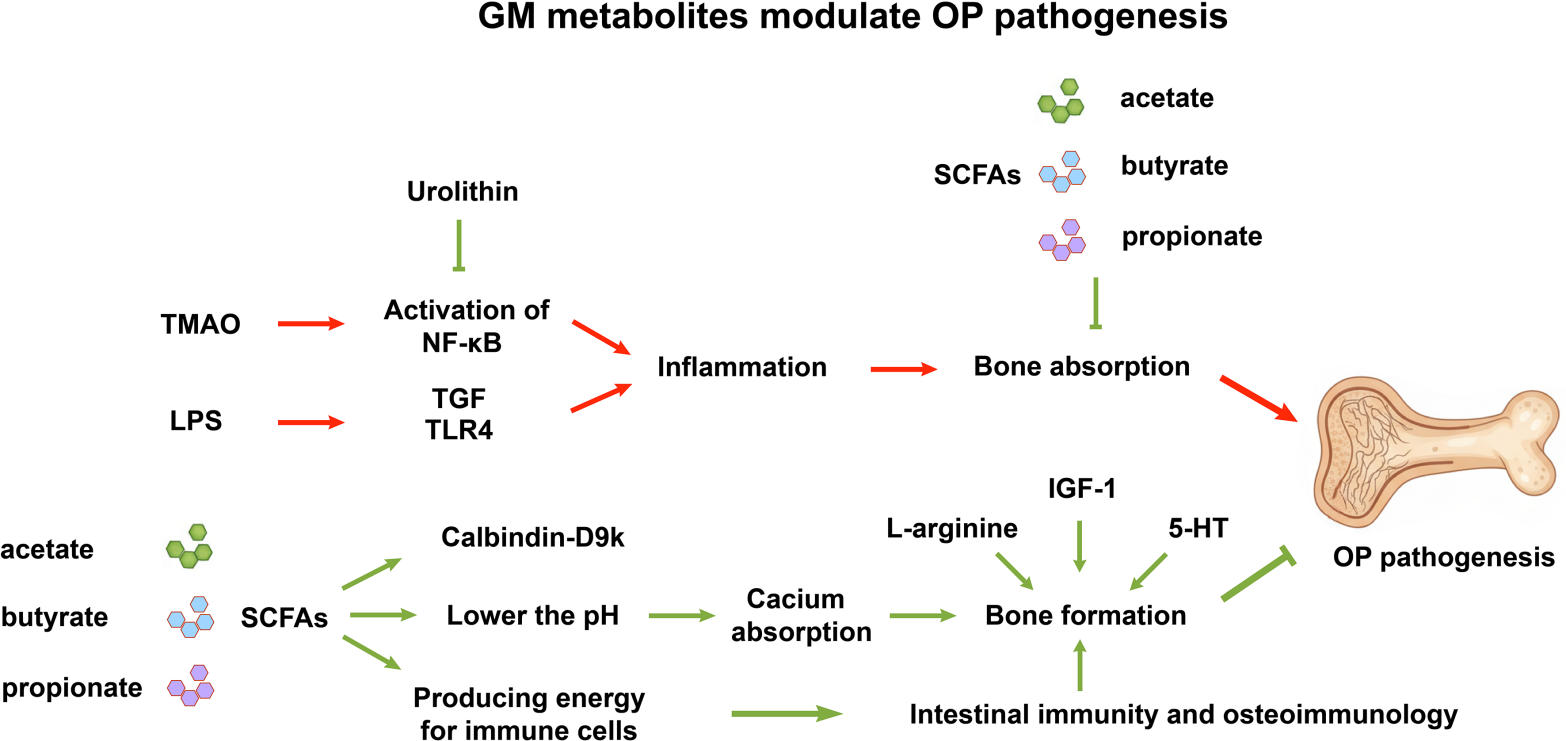
Modulation of osteoporosis (OP) pathogenesis by gut microbiota-derived metabolites. The diagram illustrates the dual impact of microbial metabolites on bone remodeling. Pathogenic factors such as TMAO and LPS activate inflammatory pathways (NF-κB, TGF, TLR4), leading to inflammation and subsequent bone resorption, thereby promoting OP development (red arrows). Conversely, SCFAs, including acetate, butyrate, and propionate, exert dual beneficial effects on bone by inhibiting bone resorption while simultaneously promoting bone formation. GM-derived L-arginine, 5-HT, and IGF-1 promote bone formation. In addition, urolithin inhibits bone resorption by suppressing NF-κB activation. Mechanisms include the upregulation of Calbindin-D9k and pH reduction to enhance calcium absorption, providing energy for immune cells, and modulating intestinal immunity and osteoimmunology (green arrows). GM, gut microbiota; TMAO, Trimethylamine N-oxide; LPS, Lipopolysaccharides; NF-κB, nuclear factor kappa-B; TGF, transforming growth factor; TLR4, Toll-like receptor 4; SCFAs, Short-Chain Fatty Acids; 5-HT, serotonin; IGF-1, insulin-like growth factor-1.

SCFAs, such as acetate, butyrate, and propionate, are produced through the fermentation of dietary carbohydrates by the intestinal microbiota that resides commensally within the gut (57). SCFAs is crucial for the maintenance of bone mass. OVX mice showed decreased SCFAs level and high intestinal inflammation levels compared with healthy control. SCFAs could increase bone mass and strength by promoting bone formation and inhibiting bone resorption (58). SCFAs promote the absorption of calcium by increasing the expression of calbindin-D9k, a calcium-binding protein, in the intestinal epithelium (59). In addition, SCFAs could lower the pH in the intestine and increase the absorption of calcium (60). Moreover, SCFAs may regulated bone mass by affecting insulin-like growth factor-1 (IGF-1) levels, as supplementing SCFAs to the antibiotic treated mice could rebuild impaired bone mass and retore IGF-1 levels (61). SCFAs have been found crucial for the integrity of physical and chemical intestinal mucosal barriers by eliciting a response from Paneth cells and goblet cells and stimulating them to produce antimicrobial peptides (AMPs) (16, 62, 63). SCFAs also play an important role in intestinal immunity, by providing energy and affecting the activity of immune cells (64). Butyrate, a SCFA, undergoes conversion into butyryl-CoA and subsequently undergoes β-oxidation (β-OX), then participates in the tricarboxylic acid (TCA) cycle and oxidative phosphorylation (OXPHOS) (64). These metabolic processes serve to energize intestinal mucosal epithelial cells, enhance the structure of intestinal villi, suppress autophagy, and maintain intestinal homeostasis (64, 65). SCFAs can also produce energy for memory T cells, effector T cells, B-cell by glycolysis and OXPHOS, playing an important role in multiple biological processes mediated by these immune cells such as differentiation into plasma cells, metabolic changes, and antibody production (66–69) (**Figure 1**, **Table 1**).

**Table 1:** The effect of GM-derived metabolites in OP pathogenensis.

Metabolites	Effects	References
Short-chain fatty acids (SCFAs)	Promote bone formation and inhibit bone resorption; increase calcium absorption; improve intestinal barrier integrity and modulate immunity.	(57–61)
Butyrate	Energizes intestinal epithelial cells, enhances villi structure, suppresses autophagy, and maintains intestinal homeostasis.	(64, 65)
L-citrulline/L-arginine	Improve bone mechanoresponsiveness by activating a nitric oxide-calcium positive feedback loop in osteocytes.	(70)
Urolithin A	Inhibits RANKL-induced osteoclastogenesis and reduces expression of inflammatory cytokines (e.g., IL-6, TNF-α) in osteoclasts.	(71)
Urolithin B	Prevents bone loss by suppressing the ERK/NF-κB signaling pathway.	(72)
Trimethylamine N-oxide (TMAO)	Inversely correlates with BMD; accelerates bone loss via NF-κB activation in BMSCs; predictive biomarker for OP in overweight/obese and T2DM patients.	(73)

In addition to the aforementioned beneficial metabolites, certain other metabolites also contribute to the protection against OP. Moreover, research has demonstrated that GM depletion significantly compromises the skeletal system’s capacity to adapt to mechanical stress (70). The microbial synthesis of L-citrulline and its subsequent conversion to L-arginine have emerged as crucial modulators of bone mechanoadaptation. Administration of these metabolites was found to improve the bone’s mechanoresponsiveness in mice, which was due to the activation of a nitric oxide-calcium positive feedback loop within osteocytes (70). Urolithin A, a biologically active metabolite produced by the GM, mitigates OVX-induced OP by inhibiting osteoclastogenesis triggered by RANKL and reducing the expression of inflammatory cytokines such as interleukin (IL)-6 and tumor necrosis factor-α (TNF-α) in osteoclasts (71), and Urolithin B, prevent bone loss by suppressing ERK (extracellular regulated protein kinases)/NF-κB (nuclear factor kappa-B) signaling pathway (72) (**Figure 1**, **Table 1**).

Certain bacterial metabolites can serve as predictive indicators for bone loss and OP. Trimethylamine N-oxide (TMAO), a metabolite derived from GM, exhibits an inverse relationship with BMD and a direct correlation with the occurrence of osteoporotic fractures in patients with type 2 diabetes mellitus (T2DM) (73). In individuals who are overweight or obese, circulating levels of TMAO may serve as useful biomarkers for the early detection of OP (74). Furthermore, another study indicates that elevated TMAO levels demonstrate a strong negative association with the severity of BMD loss in OP (75). By modulating the function of BMSCs via the activation of the NF-κB signaling pathway, TMAO accelerates bone loss and thereby facilitates the progression of OP (75) (**Figure 1**, **Table 1**).

### Gut hormone

The GM is increasingly regarded as a full-fledged endocrine organ due to its ability to secrete a wide array of bioactive molecules (76, 77). An important mechanism by which GM affects the systemic health is the modulation of bone metabolism through regulation of several hormones. These include parathyroid hormone (PTH), IGF-1, serotonin (5-HT), melatonin, peptide YY (PYY), and the incretin hormones glucose-dependent insulinotropic polypeptide (GIP), glucagon-like peptide 1 (GLP-1), and glucagon-like peptide 2 (GLP-2) (78, 79).

Interestingly, many gut-derived hormones are secreted in a rhythmic pattern rather than a steady state. The release of gut-derived incretin hormones follows a rhythm driven by dietary intake, host circadian clocks, and environmental cues. This hormonal rhythm is paralleled by the GM itself, which shows significant 24-hour rhythmicity in its composition and metabolic activity (80), suggesting a tightly coordinated host-microbiome crosstalk. Indeed, circadian rhythms are critical for bone homeostasis, as reflected by the diurnal fluctuations in bone remodeling markers from mice to humans (81).

Among the hormones regulated by GM, several have direct and specific effects on bone. For instance, GM can regulate PTH levels, thus protecting against bone resorption and promoting bone formation (82). Similarly, GM can induce IGF-1 expression—a growth factor known to promote the differentiation of osteoblasts, osteoclasts, and chondrocytes—which further stimulates bone formation (61). Moreover, GM modulates gut-derived serotonin (5-HT), a molecule that has direct contact with bone cells and has been suggested as a regulator of bone mass (83, 84).

A particularly significant GM-derived metabolite is melatonin, generated from tryptophan. Beyond its systemic role, melatonin can also modulate the balance of M1/M2 macrophage, reduce serum pro-inflammatory cytokines, and restore gut-barrier function (85). For bone, melatonin is also an crucial endogenous regulator of bone metabolism by stimulating osteoblast differentiation, inhibiting osteoclast differentiation, and inducing apoptosis in mature osteoclasts (86, 87). Conversely, OP has been linked with reduced melatonin production from the GM. Importantly, supplementation with melatonin not only alleviates clinical symptoms related to OP, but also inhibits GM dysbiosis, promotes the function of GM, increases SCFA levels, reduces trimethylamine N-oxide (TMAO), and preserves intestinal barrier integrity (85) (**Figure 1**).

The inter-relationship between GM and hormones presents another layer of complexity in postprandial bone regulation. One study suggests that GIP (secreted from intestinal K cells), GLP-1, and GLP-2 (secreted from L cells) are the main mediators of postprandial bone turnover (88). However, another study revealed a distinct role for GLP-2. While parenteral administration of GIP or GLP-1 did not lower serum levels of s-CTX (a marker of bone resorption), GLP-2 caused a dose-dependent reduction of s-CTX. This indicates that GLP-2, rather than GIP or GLP-1, plays the major role in the early postprandial suppression of bone resorption (89).

### Intestinal permeability and leaky gut

**Figure 2:**
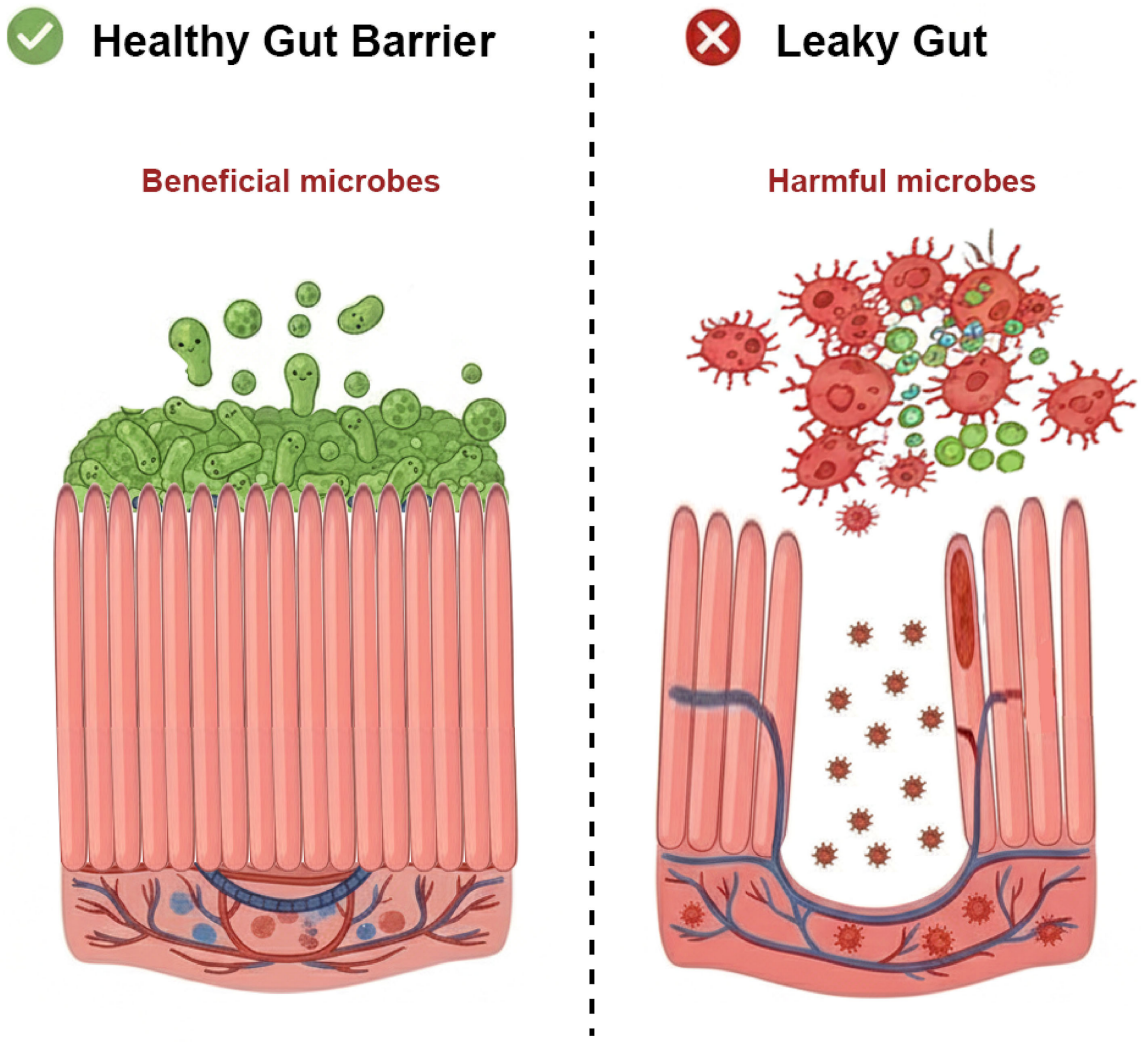
Comparison of healthy gut barrier and “leaky gut” phenotype. The left panel illustrates a healthy intestinal barrier characterized by a balanced community of beneficial microbes, a thick and intact mucus layer, and tight junctions connecting epithelial cells, which collectively prevent pathogen invasion. The right panel depicts a “leaky gut” state associated with dysbiosis (dominance of harmful microbes), disrupted tight junctions, and a compromised mucus layer. This loss of integrity facilitates the translocation of pathogenic bacteria and inflammatory factors into the systemic circulation, triggering systemic inflammation.

**Figure 3:**
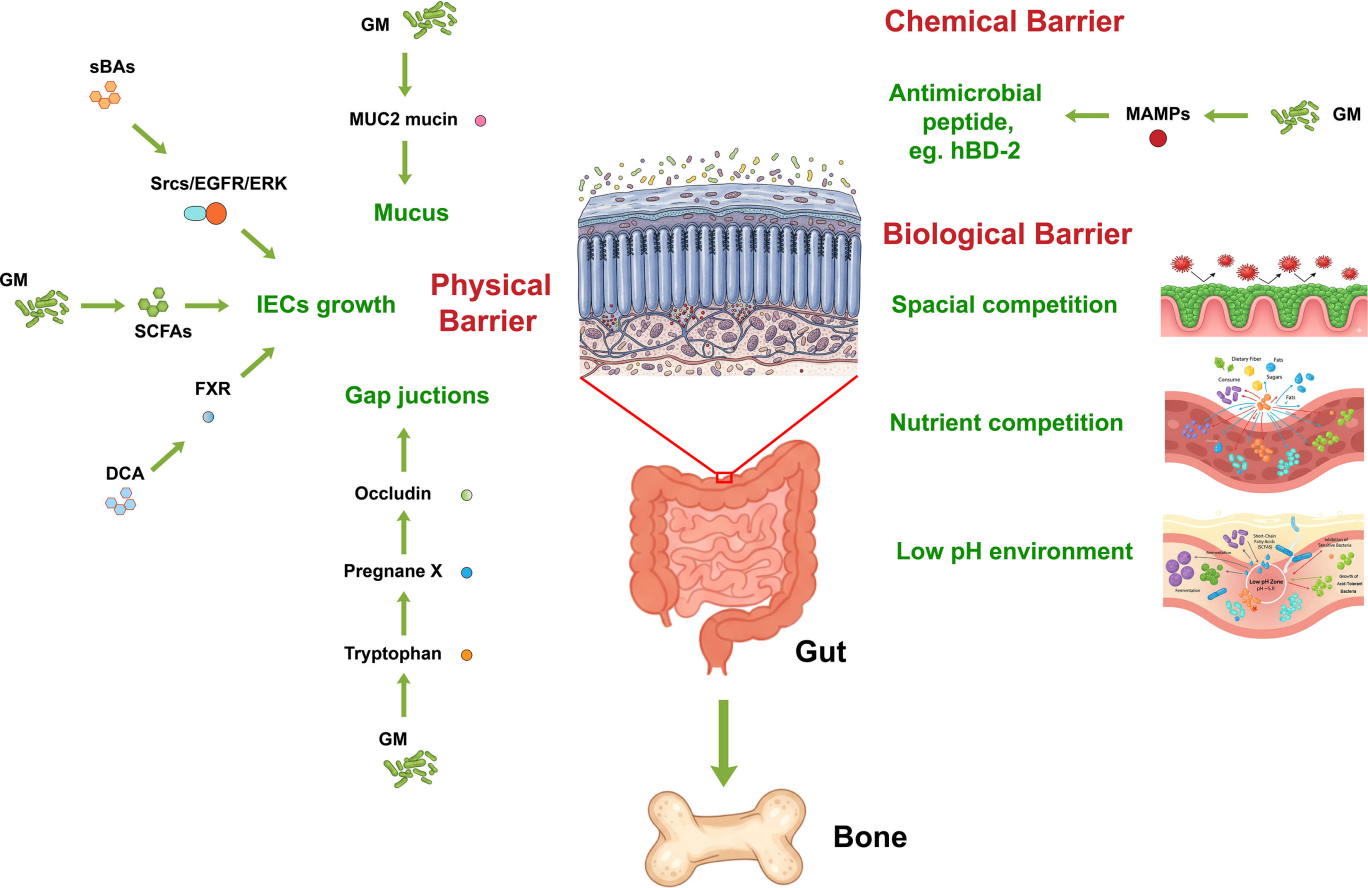
Mechanisms by which healthy gut microbiota maintains intestinal barrier integrity. A healthy GM reinforces the intestinal barrier through multiple ways: (1) Physical Barrier: GM-derived metabolites (DCA, sBAs, SCFAs, Tryptophan) activate key receptors (FXR, Pregnane X) and signaling cascades (Srcs/EGFR/ERK) to promote mucin production (MUC2), IEC growth, and the expression of tight junction proteins (Occludin). (2) Chemical Barrier: Beneficial GM produce antimicrobial peptides like hBD-2 via MAMPs. (3) Biological Barrier: Beneficial GM suppresses pathogens via spatial competition, nutrient competition, and maintenance of a low pH environment, collectively protecting the bone by preventing systemic inflammation. GM, gut microbiota; DCA, deoxycholic acid; sBAs, secondary bile acids; SCFAs, Short-Chain Fatty Acids; FXR, farnesoid X receptor; EGFR, epidermal growth factor receptor; ERK, extracellular signal-regulated kinase; IEC, intestinal epithelial cell; MAMPs, microbial-associated molecular patterns.

Alterations in GM and impairment of the intestinal barrier contributed to the development of OP (90). The intestinal barrier is crucial for maintaining intestinal integrity and preventing bacteria and their pro-inflammatory toxins from leaking into the bloodstream. Reduced barrier function with increased intestinal permeability is usually occurred during OP, leading to the migration of bacteria and their harmful metabolites into the circulation, which is called leaky gut (91) (**Figure 2**). Healthy GM helps maintain the intestinal barrier while abnormal GM could damage the intestinal barrier, aggravating leaky gut and OP process. The intestinal barrier is a multifaceted structure, comprising physical, chemical, and biological barriers (92). The GM plays a pivotal role in modulating intestinal barrier function across these distinct levels. Specifically, GM predominantly regulates the integrity of the intestinal barrier through its metabolites, which exert profound influences on the metabolism, proliferation, apoptosis, and genetic alterations of epithelial cells, although there are additional interactions between GM and intestinal epithelial cells that collectively contribute to the regulation of the intestinal barrier (**Figure 3**).

Three key bacterial metabolites—SCFAs, sBAs, and tryptophan derivatives—have been recognized as essential factors in preserving the integrity of the gut epithelium (93). There are several G protein coupled receptors (GPCRs) for SCFAs on the cell membrane of intestinal epithelial cells (IECs), such as GPR41/43 and GPR 109a (57). SCFAs preserve the integrity of the gut epithelium by activating these receptors. For example, the GPR41/43 help clear pathogenic bacteria by increasing the production of cytokines and chemokines (94). Butyrate induces the expression of the inflammatory factor IL-18 through the activation of GPR109a (95). Butyrate activates peroxisome proliferator-activated receptor γ (PPAR-γ) in colonic epithelium and inhibits the proliferation of *Enterobacteriaceae* bacteria by hampering respiratory electron acceptors (96). SCFAs (especially butyrate) significantly upregulate the gene expression of antimicrobial peptides, particularly β-defensins (e.g., hBD-2), which constitute a critical component of the chemical barrier (97). In addition, sodium butyrate stimulate an increase in the production and secretion of mucus by goblet cells, which strengthen the physical barrier of intestine (98). Moreover, SCFAs serve as an energy source that stimulates the growth of IECs and also regulate the immunological functions of IECs by influencing the expression of genes related to cytokines and mucins. sBAs produced by the GM have an impact on the activity and proliferation of IECs. For instance, taurine-conjugated bile acids stimulate the growth of IECs by activating Src, the epidermal growth factor receptor (EGFR), and ERK pathways. Conversely, their unconjugated secondary counterpart, DCA, hinders IEC proliferation through the activation of the farnesoid X receptor (FXR) (99, 100). Dietary tryptophan is converted into indole by tryptophanase, an enzyme produced by the GM. This indole compound has the ability to promote the expression of cell-junction-associated molecules, such as occludin and claudins, by activating the pregnane X receptor, thereby enhancing the integrity of intestinal epithelial cells (101) (**Figure 3**).

In addition to these metabolites mentioned above, GM regulate intestinal barrier through several other mechanisms. The colonic epithelium is enveloped by a two-layered mucus framework, centered around MUC2 mucin, which serves as a crucial physical barrier (102). The inner mucus layer, characterized by its density and adherence, remains impermeable to the GM, thus ensuring a bacteria-free environment on the epithelial cell surface. Conversely, the outer, more permeable mucus layer acts as a habitat for the commensal GM (102). *Lactobacillus rhamnosus* GG and *Akkermansia* spp. exhibit the ability to stimulate goblet cells, inducing mucin secretion (predominantly MUC2) (103, 104). This secretion process augments the mucus layer’s thickness and continuity, thereby fortifying the physical barrier. A multitude of symbiotic bacteria, encompassing lactobacilli and bifidobacteria, possess the capability to synthesize bacteriocins endowed with direct antibacterial or bactericidal attributes (105). These symbiotic microbiota, primarily through the microbial-associated molecular patterns (MAMPs) adorning their surfaces, such as lipopolysaccharides (LPS), peptidoglycans, flagellin, and cell wall dipeptides (106), directly trigger the expression of antimicrobial peptides. These peptides include defensins, members of the RegIII family proteins, and calprotectin (107). The induction process transpires via interactions with pattern recognition receptors (PRRs), including Toll-like receptors (TLRs) and nucleotide-binding oligomerization domain-like receptors (NLRs), which are expressed on both intestinal epithelial cells and immune cells, including dendritic cells (108, 109). By doing so, it prevents these pathogens from eroding the mucus layer and compromising the epithelial cell integrity (110). Furthermore, A healthy symbiotic microbiota effectively inhibits the overgrowth and colonization of foreign pathogens or endogenous opportunistic pathogens by occupying ecological sites (spatial competition), consuming limited nutrients (nutritional competition), and creating an environment that is unfavorable for pathogen growth (such as reducing pH through acid production). This directly reduces the attack of pathogens on physical and chemical barriers. For example, a well-balanced microbial community structure can effectively curb the proliferation of Proteobacteria, which secret mucin-degrading enzymes like proteases and glycosidases and damage the intestinal barrier (111). Moreover, beneficial GM exerts antimutagenic activity effect, which helps maintain the intestinal barrier by mitigating DNA damage in epithelial cells (58). Specific lactic acid microbiota species have the capability to bind potent mutagens, such as pyrolyzates (112, 113) and heterocyclic amines (114), within the gut, thereby reducing the mutagenic potential of these compounds (**Figure 3**).

### Intestinal immunity and osteoimmunology

Osteoimmunology disorder is related to several bone diseases such as PMO and inflammatory bone loss (32, 115). The GM could influence the inflammatory milieu by modulating T cells, which in turn affects the production of immune mediators and inflammatory cytokines, ultimately stimulating osteoclastogenesis and leading to bone loss (116, 117). Previous studies showed that GM could modulate innate immunity and adaptive immunity, involving several immune cells such as T cells, dendritic cells, NK cells and Monocytes/macrophages (16). Melanoma patients who have a high abundance of beneficial microbiota, such as *Clostridiales*, *Ruminococcaceae*, and *Faecalibacterium* spp., exhibit enhanced antigen presentation and improved functionality of effector CD4+ and CD8+ T cells in their peripheral blood (118). *Faecalibacterium* elevated the proportion of CD4+ T cells and increased serum CD25 production, while simultaneously decreasing the proportion of Treg cells in peripheral blood (119). *Bacteroides fragilis* promotes IL-12-dependent Th1 immune responses by enhancing the migration of dendritic cells (DCs) in the lamina propria (120). Research has demonstrated that animals raised in a germ-free environment possess immature mucosal immune systems characterized by underdeveloped gut-associated lymphoid tissue. Additionally, GF mice exhibit a decreased count of CD4+ T cells in their spleens, along with a reduction in both the number and size of germinal centers within the spleen, implying that the GM plays a crucial role in modulating systemic immunity (93, 121) (**Table 2**).

**Table 2:** The role of GM and their metabolites in intestinal immunity and osteoimmunology.

GM or their metabolites	Effects	References
*Faecalibacterium* spp.	Elevates CD4+ T cells proportion, increases serum CD25 production, decreases Treg cells in peripheral blood.	(117)
*Bacteroides fragilis*	Promotes IL-12-dependent Th1 immune responses by enhancing dendritic cell migration in lamina propria.	(118)
SCFAs	Stimulate GPCRs, inhibit HDACs, enhance gut epithelial integrity, increase Tregs, reduce inflammatory cytokines.	(57, 120)
Butyrate	Suppresses proinflammatory mediators in macrophages, inhibits dendritic cell maturation, activates GPR109a signaling, promotes Tregs and IL-10^+^ T cells accumulation.	(93, 121, 122)
*Clostridiales, Ruminococcaceae*	Enhance antigen presentation and improve functionality of effector CD4^+^ and CD8^+^ T cells.	(116)

GM mainly affects immune cells through bacterial components and metabolites. SCFAs impact intestinal immunity in various ways, including stimulating GPCRs, inhibiting histone deacetylases and gene transcription, and eliciting intracellular metabolic alterations (57). SCFAs exert their modulatory effects at numerous levels, involving promoting gut epithelial integrity, increasing the number and function of Foxp3+ regulatory T-cells (Tregs) by inducing TGF-β production in epithelial cells, and reducing the expression of various inflammatory cytokines, ultimately altering mucosal homeostasis (57, 122). Butyrate sustains reduced immune responsiveness to commensal bacteria by suppressing the expression of proinflammatory mediators in macrophages (123) and inhibiting dendritic cell (DC) maturation (124). This effect is mediated through its histone deacetylase (HDAC)-inhibitory activity. Additionally, butyrate activates GPR109a signaling, which confers anti-inflammatory properties to macrophages and DCs, thereby promoting the accumulation of Tregs and IL-10-producing CD4^+^ T cells in the colon (95) (**Table 2**).

OVX-induced bone loss has been linked to increased production of osteoclastogenic cytokines by bone marrow cells, including T cells and stromal cells. CD40L, a T-cell costimulatory molecule on T cell membrane, is required for OVX-induced stromal cells proliferation. T cells or CD40L depletion in mice could protect mice from OVX-induced bone loss. This study demonstrated that CD40L mediated interaction between T cells and stromal cells plays a vital role in the dysregulation of osteoblastogenesis and osteoclastogenesis in OVX-induced OP (125). Furthermore, OVX leads to an enhanced expansion and migration of Th17 cells and TNF+ T cells from the intestine to the bone marrow, a process that is dependent on GM. Inhibiting the egress of Th17 cells and TNF+ T cells from the gut or their infiltration into the bone marrow can alleviate OVX-induced bone loss and OP development (126, 127). This study emphasized the potential of intestinal T cells as a target for treating OP, suggesting that blocking the migration of intestinal T cells may represent a viable therapeutic approach for OP treatment.

## Potential solutions for treating OP by intervening in GM

### Probiotics

**Figure 4:**
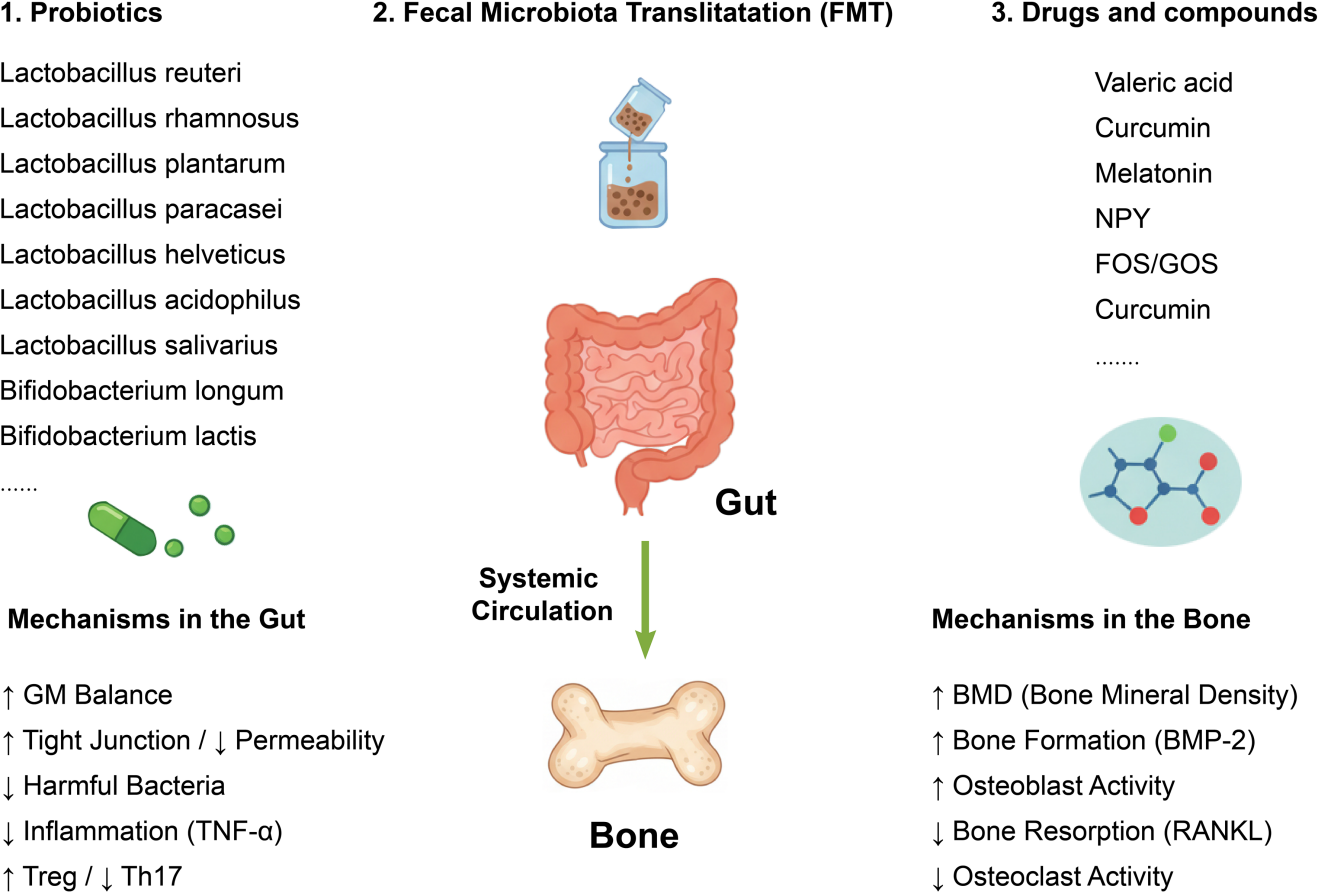
Therapeutic strategies targeting the GM for OP management. This schematic summarizes therapeutic interventions, including Probiotics (e.g., L. reuteri, B. longum), Prebiotics (FOS/GOS), bioactive compounds (e.g.,Valeric acid, Curcumin, Melatonin), and FMT. Mechanisms in the Gut: These strategies restore GM balance, enhance tight junctions, decrease permeability, and shift the immune profile (reduced TNF-α/Th17, increased Treg cells). Mechanisms in the Bone: these gut-level improvements lead to increased BMD and osteoblast activity (Bmp-2), alongside decreased osteoclast activity and bone resorption (RANKL inhibition), ultimately mitigating OP. FOS, fructooligosaccharides; GOS, galactooligosaccharides; TNF-α, tumor necrosis factor-α; BMD, Bone Mineral Density; FMT, Fecal Microbiota Transplantation; RANKL, Receptor Activator of Nuclear Factor-κB Ligand.

Many probiotics and prebiotics are reported to be beneficial for bone homeostasis and prevent OP development. By altering the composition of the gut microbiome, probiotics have the potential to boost the gene expression of Bmp-2 and Sparc, which are two crucial factors that promote the differentiation of BMSCs into osteoblasts, thereby enhancing bone mass that have been diminished due to OVX (128). Probiotics could lower the levels of inflammatory cytokines, including IL-17 and TNF-α, by reducing the proportion of Th17 cells (129, 130). Additionally, they exert anti-inflammatory effects by enhancing the number of Treg cells and promoting the production of anti-inflammatory factors, such as IL-4 and IL-10 (129, 130). Moreover, probiotics could reduce the intestinal permeability and leaky gut (131), hence reduce the secretion of harmful factors such as LPS, TNF-α and RANKL, ultimately ameliorate bone loss and OP development (129, 132, 133) (**Figure 4**).

The specific prebiotics that have been reported previously are as follows:

*Lactobacillus reuteri* has demonstrated the ability to enhance bone mineral density in OVX murine models (134) and male mice (135, 136) but have no significant effect on the intact female mice (136). Furthermore, this bacterium is postulated to have anti-TNF-α activity, thereby modulating bone metabolism via the immune system (135). This speculation was supported by another study, suggesting *Lactobacillus rhamnosus* inhibited bowel inflammation and prevented bone loss by downregulating the expression of RANKL and TNF-α (33). This study also found that estrogen deficiency-induced bone loss was dependent on GM and probiotics supplement helps prevent OP (33). In postmenopausal women, taking *Lactobacillus reuteri* orally has been shown to elevate tibial bone density (137) and boost circulating levels of vitamin D (138). Both *Lactobacillus rhamnosus* and *Lactobacillus plantarum* have the capacity to increase serum vitamin D levels by enhancing the expression of vitamin D receptor in enterocytes of both mice and humans (139). This research also indicated that vitamin D plays a role in maintaining immune homeostasis, at least in part, through its interaction with the GM (139). However, a recent randomized clinical trial involving 239 women in the early stages of postmenopausal life found that daily supplementation with *Limosilactobacillus reuteri* ATCC PTA 6475, over a period of two years, had no discernible impact on bone loss or bone turnover rates (140). The study also noted an intriguing interaction between BMI and the treatment effect, which requires further research and exploration (140) (**Figure 4**, **Table 3**).

**Table 3:** Probiotics against OP.

Probiotics	Effects	References
*Bifidobacterium longum*	Upregulates Sparc and Bmp-2 genes, increases bone mass density in OVX rats.	(60, 126)
*Lactobacillus reuteri*	Enhances BMD in OVX and male mice; anti-TNF-α activity; increases tibial bone density and vitamin D in postmenopausal women.	(132–136)
*Lactobacillus rhamnosus*	Inhibits bowel inflammation, prevents bone loss by downregulating RANKL and TNF-α; increases vitamin D receptor expression.	(33, 137)
*Lactobacillus plantarum*	Increases serum vitamin D levels; mitigates glucocorticoid-induced OP; alters gut microbiota and serum metabolites; suppresses RANKL/OPG pathway.	(60, 137, 140, 141)
*Lactobacillus paracasei*	Reduces IL-1β and TNF-α levels, inhibits osteoclastogenesis in OVX models.	(139)
*Lactobacillus helveticus*	Reduces IL-1β and TNF-α levels, inhibits osteoclastogenesis in OVX models.	(139)
*Lactobacillus salivarius* LI01	Prevents OP development in OVX mice.	(42)
*Lactobacillus acidophilus*	Ferments astragalus polysaccharides to promote calcium absorption and ameliorate OP.	(142)
*Bifidobacterium lactis* BL-99	Protects against colitis-induced OP by modulating gut microbiota, restoring intestinal barrier, inhibiting inflammation.	(143)
*Limosilactobacillus reuteri* ATCC PTA 6475	No significant effect on bone loss or turnover in early postmenopausal women (with BMI interaction noted).	(138)

Similar protective benefits have been reported for both *Lactobacillus paracasei* and *Lactobacillus helveticus*, as they impact osteoclastogenesis by reducing the levels of IL-1β and TNF-α in animal models that have undergone OVX (141). *Bifidobacterium longum* can also partially alleviate bone loss induced by OVX in rats by upregulating the expression of Sparc and Bmp-2 genes (128). *Lactobacillus plantarum* mitigates glucocorticoid-induced OP in rats by changing the GM composition and serum metabolic profile (142). Specifically, it notably elevates serum levels of Pyrazine and gamma-Glutamylcysteine, which are implicated in suppressing bone resorption and enhancing bone formation. There is a significant increase in the abundance of beneficial bacteria, including *Christensenellaceae_R_7_group*, *Romboutsia*, *Ruminococcus*, *UCG_005*, and *Lachnospiraceae_NK4A136_group*, whereas the abundance of harmful bacteria, such as *Desulfovibrionaceae*, is markedly reduced following *Lactobacillus plantarum* treatment (142). Moreover, *Lactobacillus plantarum NK3* and *Bifidobacterium longum NK49* alleviate OP in mice by regulating GM composition and inhibiting NF-κB-linked TNF-α expression (60). However, one study pointed out that *Lactobacillus reuteri 6475* increases bone density and in female mice only under an inflammatory condition. *Lactobacillus plantarum* LP45 suppresses the RANKL/OPG signaling pathway and protects against glucocorticoid-induced OP in rats (143). Supplementing OVX mice with *Lactobacillus salivarius* LI01, belonging to the *Firmicutes* phylum, has been found to prevent the development of OP (42). Fermentation of astragalus polysaccharides with *Lactobacillus acidophilus* promotes calcium absorption and ameliorates OP by regulating GM (144). *Bifidobacterium lactis* BL-99 protects against colitis-induced OP in mice by modulating GM, restoring intestinal barrier integrity, and inhibiting inflammation (145) (**Figure 4**, **Table 3**).

Although probiotics have shown good effects in animal models of OP, the efficacy of probiotics in treating OP may be overestimated, as there are notable differences between animal models and human disease. Typically, animal OP models are acute, accompanied by drastic metabolic and hormonal changes, which differ significantly from the natural progression of age-related OP and PMO. The role of probiotics in these contexts is closely related to the complex gut environment and specific disease background under different etiologies.

### FMT

In recent years, FMT has emerged as a novel treatment approach, primarily involving the transfer of bacteria from healthy individuals to those with GM imbalance, with the aim of reestablishing intestinal microbiota homeostasis and alleviating GM dysregulation (146). Although there is no standardized FMT strategy for changing GM composition currently, several studies have revealed the influence of FMT on the bone metabolism, intestinal barrier immune cell and inflammatory factors (147). FMT is regarded as a promising candidate for the prevention and treatment of PMO in the future, due to its role in reducing OVX-induced bone loss in mice by reestablishing the balance of GM, improving the levels of SCFAs, optimizing intestinal permeability, and suppressing the secretion of osteoclastogenic cytokines, including TNF-α and IL-1β (148, 149). *Prevotellaceae*, *Lachnospiraceae* and *Ruminococcaceae* were reported as vital components in FMT that participate in the re-establishment of OVX-induced GM disorder (149). Germ-free mice showed increased bone mass, reduced CD4+ T cells and TNF-α levels compared to normal counterparts, while FMT could restore these indexes to normal levels (117). Moreover, FMT could ameliorate glucocorticoid-induced OP (GIO) in mice, which indicated the significance of GM in the GIO process (150) (**Figure 4**).

Not all fecal samples are suitable for FMT in the treatment of OP. For instance, feces from aged individuals or those with OP may the condition. While one study reported that transplanting GM from aged mice into young, healthy mice led to a reduction in lean mass without affecting bone mass (151), another study demonstrated that transferring fecal matter from aged osteoporotic rats to younger ones induced GM dysbiosis and promoted OP (90). They believe the altered GM and compromised intestinal mucosal barrier play pivotal roles in this condition (90). To date, no consensus has been reached on the criteria for appropriate feces selection.

### Drugs and compounds

Various drugs and compounds have demonstrated the ability to slow down the progression of OP by modulating the GM (152). NPY is a multi‐functional neuropeptide that is mainly secreted by hypothalamus and is abundant in brain, gut, and bone (153). The expression of NPY, which will increase with aging and OP, together with its receptor Y1R, plays a strong part in bone metabolism. NPY decreased bone formation while its receptor Y1Ra, a GPCR, could reduce bone loss in OVX rats (27). NPY and Y1Ra prevent OP by changing the compositions of GM and modulating intestinal permeability (27). In mice, fructooligosaccharides (FOS)/galactooligosaccharides (GOS) mitigates bone loss induced by a high-fat diet by reversing GM imbalance, reducing intestinal permeability, and alleviating systemic inflammation (154). A randomized controlled trial (RCT) has demonstrated that daily supplementation with blackcurrant for six months can ameliorate bone loss in 51 postmenopausal women (155). This beneficial effect may be attributed to its ability to modify the composition of GM and inhibit the production of osteoclastogenic cytokines (155). The administration of 2’-fucosyllactose reversed bone loss and microstructural damage in naturally aging mice by mitigating colonic inflammation, gut leakage, and tight-junction damages (156). Additionally, 2’-fucosyllactose exhibited the ability to inhibit M1 macrophages polarization, rejuvenate GM diversity, restore the populations of *Bifidobacterium* spp., *Prevotellaceae*, and *Akkermansia* spp., while simultaneously impeding the growth of *Stenotrophomonas* spp (156) (**Figure 4**, **Table 4**).

**Table 4:** Drugs or compounds in treating OP by affecting GM.

Drugs or compounds	Effects	References
Neuropeptide Y (NPY) & Y1Ra	Changes GM composition and intestinal permeability, reduces bone loss in OVX rats.	(27, 150)
Fructooligosaccharides (FOS)/Galactooligosaccharides (GOS)	Reverses GM imbalance, reduces intestinal permeability, alleviates systemic inflammation, mitigates high-fat diet-induced bone loss in mice.	(152)
Blackcurrant	Modifies GM composition, inhibits osteoclastogenic cytokines, ameliorates bone loss in postmenopausal women (RCT).	(153)
2’-Fucosyllactose	Mitigates colonic inflammation and gut leakage, inhibits M1 macrophage polarization, restores beneficial bacteria (e.g., Bifidobacterium, Akkermansia), reverses bone loss in aging mice.	(154)
Valeric acid	Decreases bone resorption, improves bone microstructure in OVX mice; reduces pro-inflammatory RELA, increases anti-inflammatory IL-10.	(30)
Butyric acid	Restores intestinal barrier, increases Treg cells, reverses bone loss induced by chronic Pb exposure.	(155)
Epigallocatechin gallate	Ameliorates OP in rats by modulating GM and serum metabolites.	(156)
Microbial tryptophan metabolites	Restore intestinal AhR-mediated gut-bone signaling, mitigate OVX-induced OP.	(157)
Phytosterols	Mitigate OP in OVX-induced mice by modulating GM.	(158)
Epimedium brevicornu Maxim decoction	Attenuates OP through NLRP3/Caspase-1/IL-1β pathway and modulates GM communities.	(159)
Quinoa	Alters GM composition, restores intestinal barrier, ameliorates bone microstructure and metabolism in OVX rats.	(160)
Moringa oleifera leaf	Ameliorates OP in OVX rats by altering GM and MAPK signaling pathway.	(161)
Ziyuglycoside II	Reduces systemic inflammation, fortifies intestinal barrier, modulates GM, increases SCFAs (propanoic and acetic acid), mitigates bone loss in OVX mice.	(162)
Indole propionic acid	Suppresses osteoclast formation by activating pregnane X receptor; derived from gut Clostridium sporogenes.	(163)
Curcumin	Mitigates bone loss by modulating GM composition and serum metabolome.	(164)
Pueraria lobata exosome-like nanovesicles (PELNs)	Reduce harmful GM strains, degrade TMAO, promote BMSC differentiation, increase autophagy in OVX rats.	(165)
Gold nanospheres (GNS)	Regulate GM diversity and composition, reduce TMAO metabolites, inhibit TNF-α and IL-6, prevent OVX-induced OP.	(166)
Propolis nanoemulsions (PNEs)	Modulate GM composition and metabolites, restore intestinal barrier, decrease Streptococcus, increase L-arginine, inhibit osteoclasts, promote osteoblasts.	(167)
Clostridium sporogenes-encapsulated silk fibroin hydrogel	Prevents OVX-induced bone loss.	(163)

Valeric acid, a metabolite derived from the GM, exhibited a positive correlation with BMD and was causally downregulated by *Bacteroides vulgatus*, a bacterium associated with decreased BMD. Valeric acid was observed to decrease bone resorption and improve bone microstructure in OVX mice. Mechanistically, valeric acid reduced the production of the pro-inflammatory RELA protein and improved the expression of anti-inflammatory IL-10 mRNA, leading to inhibited maturation of osteoclast-like cells and enhanced osteoblast maturation *in vitro* (30). Supplementation with butyric acid could reverse bone loss induced by chronic Pb exposure by restoring intestinal barrier and increasing the proportion of Treg Cells (157). Epigallocatechin gallate ameliorates OP in rats by modulating the GM and serum metabolite profiles (158). Microbial metabolites of tryptophan mitigate OVX-induced OP by restoring the intestinal aryl hydrocarbon receptor (AhR)-mediated gut-bone signaling pathway (159). Phytosterols mitigate OP in an OVX-induced mouse model by modulating the GM (160). Decoction of Epimedium brevicornu Maxim Attenuates OP through the NLRP3/Caspase-1/IL-1β pathway and modulates the abundance of GM communities (161). Quinoa could protect against bone loss in the OVX rats by altering the composition of GM and restoring the damaged intestinal barrier, thereby ameliorating bone microstructure deterioration and bone metabolism disturbances (162). Moringa oleifera leaf ameliorate OP in OVX rats by altering GM and MAPK signaling pathway (163). Ziyuglycoside II mitigated bone loss in OVX mice by reducing systemic inflammation, fortified the intestinal barrier, modulated the composition of GM and increased the levels of propanoic acid and acetic acid in SCFAs (164). Indole propionic acid derived from gut *Clostridium* sp*orogenes* could suppress osteoclast formation by activating pregnane X Receptor (165). Curcumin mitigates bone loss by modulating GM composition and the serum metabolome (166) (**Figure 4**, **Table 4**).

Advancements have also been made in the utilization of bioactive materials that target the GM for the treatment of OP. Some Exosome-like nanovesicles derived from Pueraria lobata (PELNs) have been shown to reduce the relative abundance of harmful intestinal strains by degrading GM-produced TMAO, promote the differentiation BMSCs, and increase autophagy in OVX-induced osteoporotic rats (167). Gold nanospheres (GNS) effectively prevented OVX-induced OP by regulating GM (168). Specifically, GNS significantly altered the diversity and composition of GM, resulting in a reduction in the abundance of trimethylamine-N-oxide (TMAO)-related metabolites (168). Additionally, GNS inhibited the release of osteoclastogenic and proinflammatory cytokines, such as TNF-α and IL-6, in the serum of OVX mice (168). Orally administered propolis nanoemulsions (PNEs) demonstrate remarkable anti-OP effects in an OVX mouse model by modulating the GM composition and metabolites, as well as restoring intestinal barrier function (169). A decrease in Streptococcus abundance and an elevation in the GM metabolite l-arginine play pivotal roles in this process, as they inhibit osteoclast activity and promote osteoblast function, thereby facilitating balanced bone remodeling and exerting anti-osteoporotic effects (169). *Clostridium* sp*orogenes*-encapsulated silk fibroin hydrogel system could prevent bone loss induced by OVX (165) (**Figure 4**, **Table 4**).

## Discussions

The connection between the gut and bone is profound, and GM plays a pivotal role in this axis. In this review, we present the current understanding of the role of GM in the pathogenesis of OP, with the aim of enlightening the potential therapeutic targets of GM in the treatment of OP. The GM plays a primary role in the progression of OP by producing metabolites, regulating intestinal mucosal permeability, and modifying submucosal immunity. It is important to acknowledge that primary OP is a multifactorial disorder, where bone loss and deterioration result from a confluence of factors such as aging, estrogen deficiency, reduced physical activity, nutritional deficits, immunosenescence, chronic low-grade inflammation, and genetic predisposition. Consequently, the role of the GM in bone metabolism varies across these different etiological contexts. For instance, GM dysbiosis driven by estrogen deficiency may predominantly exert its effects through modulating systemic inflammation and immune cell activity, whereas in age-related OP, GM alterations is often associated with several other age-related diseases, such as sarcopenia, nutritional malabsorption. Immunosenescence might play a more central role as well. Furthermore, the effects of GM-derived signals (e.g., SCFAs, LPS, or other metabolites) could be site-specific, potentially differing in their impact on trabecular versus cortical bone, or on axial versus appendicular skeleton, due to variations in local vascularity, mechanical loading, and cellular microenvironment. Therefore, while the GM represents a key modulator of bone homeostasis, its precise function and its contribution are likely conditioned by the predominant underlying etiology of bone loss and the specific skeletal site examined. Future studies stratifying patients by precise etiological subgroups and assessing site-specific bone outcomes will be crucial to determine the role of GM in the OP pathogenesis with different etiological contexts.

Although some questions remain unanswered and require future research, there is existing evidence supporting the therapeutic effect of probiotics, such as *Lactobacillus rhamnosus*, *Lactobacillus reuteri*, and *Bifidobacterium longum*, on OP. However, a consensus on the GM alteration related to OP is unlikely to emerge in the near future due to the variety of sequencing methods. Microbiome-based therapeutic approaches are increasingly attracting clinical interest, but application is still challenging due to the numerous, often unclear, impacts of GM on host biology. More importantly, the major changes in GM driven by life style, medication, genetics and other factors should be adequately aware of. Unfortunately, these influencing factors have been overlooked or an insufficient number of participants have been included in many studies, which confuse the readers and lead to harmful interpretations. In the study of high-dimensional and heterogeneous sequencing data with large sample size, focus on controlling confounding bias to explore the key bacteria truly affecting the pathogenesis of OP is of significance.

An important consideration is that the genomic abundance of GM could reflect their presence and population size, but does not necessarily reflect their *in situ* metabolic activity. The transcriptional activity, protein expression, and resultant metabolic output of a microbial community can be discordant with its genomic profile due to post-transcriptional regulation, environmental cues, substrate availability, and host-microbe interactions. For instance, a taxon with low abundance might be highly metabolically active and produce significant amounts of biologically relevant metabolites (e.g., SCFAs), whereas a highly abundant taxon could be in a quiescent state. This distinction is particularly relevant in the context of OP, where the functional consequences of GM shifts (e.g., production of inflammatory mediators or bone-active metabolites) are ultimately driven by microbial activity rather than mere presence. Therefore, future research integrating metatranscriptomics, metaproteomics, and metabolomics is essential to identify the functionally active GM components and their roles in bone pathophysiology, although distinguishing whether the metabolites are secreted by the GM, the host, or derived from the diet is difficult. Clarifying the sources of these metabolites will aid in understanding the extent to which the GM contributes to metabolite production.

## Conclusions

Therapies based on bacteria can supplement beneficial bacteria through oral probiotics or employ specific methods to eliminate harmful bacteria. Both approaches need rigorous animal research followed by large-scale clinical trials to establish their efficacy and safety in clinical practice. Overall, GM plays a crucial role in OP, and there is immense potential for future treatments targeting the GM in the management of OP.

## Glossary

5-HT: serotonin
AMPs: antimicrobial peptides
BAs: bile acids
BMD: bone mineral density
BMSCs: bone marrow stromal cells
β-OX: β-oxidation
CREB: cAMP response element-binding
CTX-1: C-terminal telopeptide of type I collagen
DCA: deoxycholic acid
DC: dendritic cell
EGFR: epidermal growth factor receptor
ERK: extracellular signal-regulated kinase
FMT: fecal microbiota transplantation
FOS: fructooligosaccharides
FXR: farnesoid X receptor
Gclc: glutamate-cysteine ligase catalytic subunit
GIO: glucocorticoid-induced OP
GM: gut microbiota
GNS: Gold nanospheres
GPCRs: G protein coupled receptors
GOS: galactooligosaccharides
GSH: glutathione
HDAC: histone deacetylase
IGF-1: insulin-like growth factor-1
IL-6: interleukin-6
LCA: lithocholic acid
LPS: Lipopolysaccharides
MAMPs: microbial-associated molecular patterns
NF-κB: nuclear factor kappa-B
NLRs: nucleotide-binding oligomerization domain-like receptors
OP: Osteoporosis
OXPHOS: oxidative phosphorylation
OVX: ovariectomy
P1NP: N-terminal propeptide
PELNs: Pueraria lobata
PNEs: propolis nanoemulsions
PPAR-γ: peroxisome proliferator-activated receptor γ
PRRs: pattern recognition receptors
PTH: parathyroid hormone
RANKL: Receptor Activator of Nuclear Factor-κB Ligand
RCT: randomized controlled trial
sBAs: secondary bile acids
SCFAs: short chain fatty acids
T2DM: type 2 diabetes mellitus
TCA: tricarboxylic acid
TGF: transforming growth factor
TLR4: Toll-like receptor 4
TLRs: Toll-like receptors
TMAO: Trimethylamine N-oxide
TNF-α: tumor necrosis factor-α
UDCA: ursodeoxycholic acid
